# An Interplay Between Autophagy and Immunometabolism for Host Defense Against Mycobacterial Infection

**DOI:** 10.3389/fimmu.2020.603951

**Published:** 2020-11-12

**Authors:** Seungwha Paik, Eun-Kyeong Jo

**Affiliations:** ^1^ Department of Microbiology, Chungnam National University School of Medicine, Daejeon, South Korea; ^2^ Infection Control Convergence Research Center, Chungnam National University School of Medicine, Daejeon, South Korea

**Keywords:** autophagy, immunometabolism, host defense, mycobacterial infection, AMP-activated protein kinase, mammalian target of rapamycin

## Abstract

Autophagy, an intracellular catabolic pathway featuring lysosomal degradation, is a central component of the host immune defense against various infections including *Mycobacterium tuberculosis* (Mtb), the pathogen that causes tuberculosis. Mtb can evade the autophagic defense and drive immunometabolic remodeling of host phagocytes. Co-regulation of the autophagic and metabolic pathways may play a pivotal role in shaping the innate immune defense and inflammation during Mtb infection. Two principal metabolic sensors, AMP-activated protein kinase (AMPK) and mammalian target of rapamycin (mTOR) kinase, function together to control the autophagy and immunometabolism that coordinate the anti-mycobacterial immune defense. Here, we discuss our current understanding of the interplay between autophagy and immunometabolism in terms of combating intracellular Mtb, and how AMPK-mTOR signaling regulates antibacterial autophagy in terms of Mtb infection. We describe several autophagy-targeting agents that promote host antimicrobial defenses by regulating the AMPK-mTOR axis. A better understanding of the crosstalk between immunometabolism and autophagy, both of which are involved in host defense, is crucial for the development of innovative targeted therapies for tuberculosis.

## Introduction

*Mycobacterium tuberculosis* (Mtb) causes human tuberculosis (TB), which remains a serious infectious disease worldwide (1). Mtb can counter host defenses by escaping phagolysosomal fusion, indeed residing within phagosomal structures (2, 3). Autophagy, a lysosomal degradation system that ensures homeostasis, is particularly sensitive to metabolic stress (4, 5). Autophagy is also a principal means of autonomous cellular defense, countering the Mtb-induced arrest of phagosomal maturation (6). Accumulating evidence suggests that immunometabolism is linked to regulation of the immune defense against pathogenic insults (7–12). Indeed, autophagy and immunometabolism interact extensively to control infection and inflammation (13, 14). Such crosstalk may determine the outcome of the innate effector pathways against a variety of infectious diseases, including TB.

Two serine/threonine kinases, adenosine 5′-monophosphate (AMP)-activated protein kinase (AMPK) and mammalian target of rapamycin (mTOR) kinase, play crucial roles in the integration of metabolic adaptation, autophagy, and immunometabolism in immune cells (15–17). The kinases sense intracellular metabolic status and serve as important upstream signaling regulators of immune responses, lysosomal activities, and host defenses during infections (15–17). Recent studies have highlighted the fact that transcription factor EB (TFEB) is a key mediator of the AMPK-mTOR axis (18), activating both autophagy and lysosomal biogenesis to promote innate immunity (19–21).

In this review, we discuss our current understanding of how autophagy and immunometabolism have a relationship when mounting a defense against Mtb infection. We describe recent advances in our understanding of AMPK-mTOR kinase signaling and pharmacological modulation of either or both autophagy and immunometabolism.

## Role Played by Autophagy in Mycobacterial Infection

Recent studies have highlighted the fact that a combination of metabolic, autophagic, and immune cell activities determine the outcome of Mtb infection (22, 23). Autophagy is a crucial host defense pathway targeting invasive intracellular pathogens including Mtb (24–26). In 2004, Deretic et al. found that interferon (IFN)-γ, a cytokine essential for induction of protective immunity against TB, activated macrophage autophagy to promote eradication of intracellular Mtb (27). Since that time, accumulating evidence has shown that many autophagy-activating pharmacological agents and/or small molecules trigger autophagy, leading to acidification of mycobacterial phagosomes by fusion with autophagosomes/lysosomes to restrict intracellular survival of Mtb (25, 26, 28–30).

During natural infection, Mtb translocation into the cytosol *via* ESX-1 triggers xenophagy pathway through p62-, NDP-52 (a selective autophagic receptor)–, and TBK-1–dependent pathways (31–33). In addition, the autophagy-related process LC3-associated phagocytosis (LAP) plays a role in phagosomal maturation and antimicrobial host defense (34, 35); however, Mtb CpsA, a LytR-CpsA-Psr (LCP) domain-containing protein, works to evade LAP during Mtb infection (36). The detailed mechanisms of several types of autophagy pathways in the context of mycobacterial infection have been extensively described elsewhere (25, 26, 28–30). In addition, a discussion of the Mtb effectors that induce, or allow evasion of, host xenophagy/LAP is beyond the scope of this review.

## Overview of Immunometabolism During Mycobacterial Infection

Metabolic reprogramming of innate immune cells is closely related to various cellular functions, including the production of pro-inflammatory cytokines/chemokines, autophagy activation, and mounting of antimicrobial responses to Mtb infection (22, 23, 37). It is generally thought that, upon Mtb infection, macrophages (the principal phagocytes active during infection) undergo metabolic reprogramming into M1-type macrophages in response to Mtb components or *via* Mtb phagocytosis. In these cells, pro-inflammatory molecules are upregulated and glycolysis is predominantly utilized to meet their bioenergetic and metabolic requirements, while M2-type macrophages and the non-infected/naïve cells exhibit anti-inflammatory characteristic and derive their energy from oxidative phosphorylation and fatty acid β-oxidation (FAO) (23). However, Mtb is able to perturb the metabolic switch of phagocytes that reminisce Warburg effect, a bioenergetic shift utilizing aerobic glycolysis, to facilitate bacterial pathogenesis *via* enhancement of intracellular bacterial survival and persistence (38). To support this, a recent study showed that Mtb infection restricts glycolysis and interleukin (IL)-1β production by upregulating miR-21, thereby favoring intracellular Mtb growth (39). Given the previous reports on how miR-21 inhibits autophagy in a variety of scenarios (40–42), it would be interesting to explore whether miR-21 suppresses autophagy to potentiate immunopathogenesis during Mtb infection. During chronic Mtb infection, the mitochondrial metabolism of CD8+ T cells becomes defective; mitochondrial dysfunction increases (37). It remains to be determined whether aerobic glycolysis is up- or down-regulated during chronic Mtb infection. Importantly, metformin, an activator of AMPK and autophagy, improved Mtb-specific CD8+ T cell immunity by rescuing T cell bioenergetics (37), although autophagy was not investigated in the context of such metformin-induced reinvigoration. It would be useful to clarify the function and mechanism of autophagy in the regulation of immunometabolic remodeling, and how this impacts host defenses during the various stages of Mtb infection.

It is also intriguing that Mtb-infected host cells exhibit different aspects of metabolic shift depending on the virulence of Mtb strains. A previous study revealed that genes associated with inflammation and metabolism were downregulated in virulent H37Rv strain when compared to attenuated H37Ra strain infection in human alveolar macrophages (43). In other studies, Mtb infection compromised metabolic reprogramming, while infection with the BCG or dead Mtb upregulated glycolytic flux in human monocyte-derived macrophages (44). Multidrug-resistant Mtb strains preferentially induce IFN-β that limits IL-1β induction, resulting reduced aerobic glycolysis when compared to drug susceptible Mtb strains (45). Since infections with live, virulent Mtb decelerate the metabolic switch shifting to glycolytic pathway of host cells, the future studies unveiling the molecular mechanisms controlled by mTOR and/or AMPK, which are master regulators of immunometabolism, in terms of virulence of Mtb strains will accelerate the development of anti-mycobacterial therapeutics.

Mtb modulates (interferes with) host cell lipid metabolism during infection. Mtb induces numerous proteins involved in FAO; the lipids yield energy and act as building blocks for membrane synthesis (46). It remains to be determined whether FAO may suppress the host defense against Mtb infection. Either FAO blockade or a deficiency of the mitochondrial fatty acid transporter carnitine palmitoyltransferase 2 reduces the burden of Mtb both *in vitro* and *in vivo*. Mechanistically, FAO inhibition enhances mitochondrial reactive oxygen species (mitoROS) production, promoting NADPH oxidase activity and xenophagy in macrophages infected with Mtb (47). The activation of the peroxisome proliferator-activated receptor (PPAR)-α enhances an anti-mycobacterial immune defense by promoting lipid catabolism, and autophagy *via* TFEB (48). Although PPAR-α activation promotes the transcriptional activation of genes involved in FAO in macrophages (48), it should be clarified whether PPAR-α–mediated FAO drives anti-mycobacterial effects. Given the findings that blockade of FAO contributes to the antimicrobial host defense (47), future studies are needed to elucidate how the lipid metabolic reprogramming is linked to host autophagy/lipophagy to further regulate host defense against Mtb infection.

**Figure 1:**
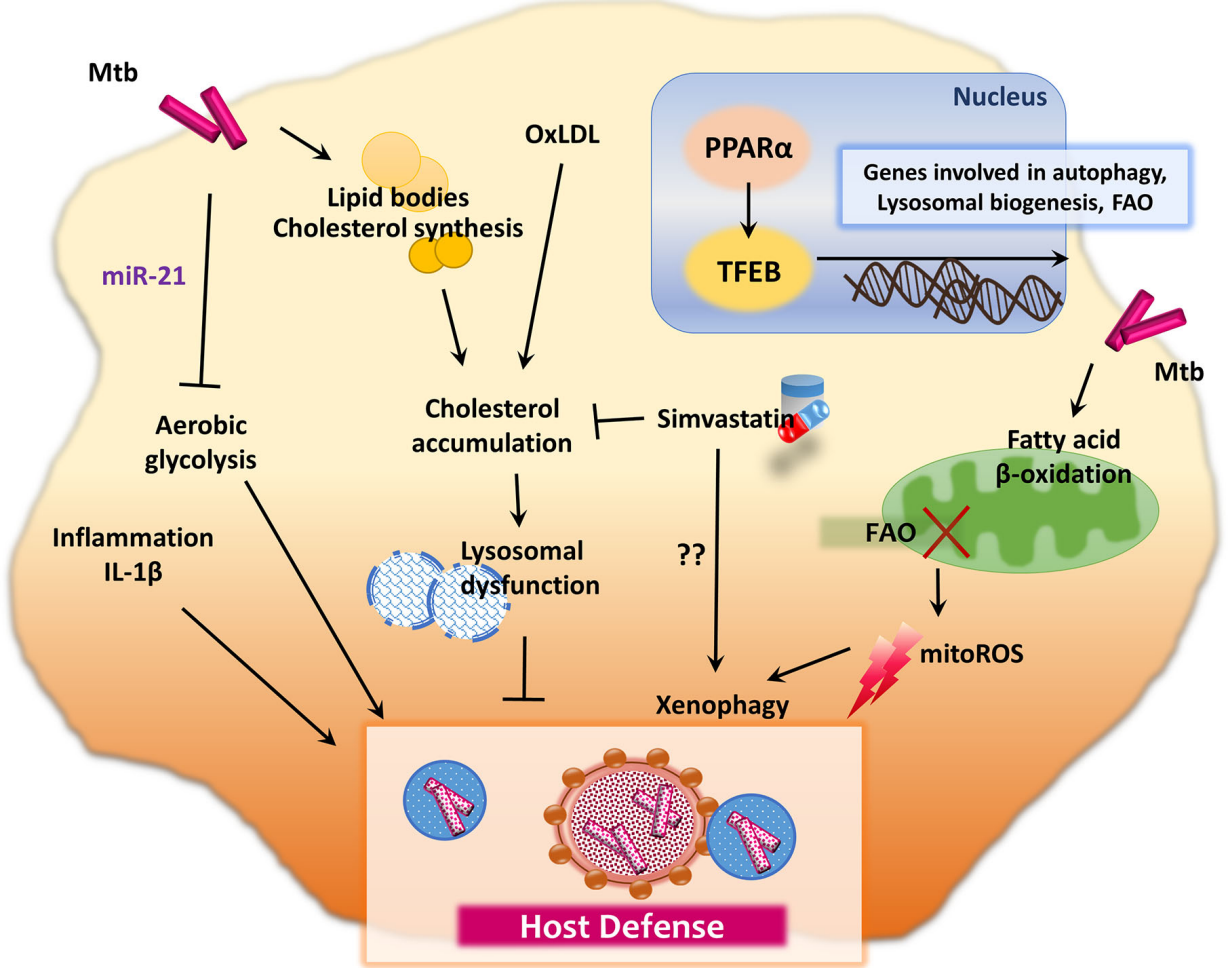
Immunometabolic pathway during mycobacterial infection. Mtb intervene in host cell lipid metabolism for its own intracellular survival. During the metabolic reprogramming process, innate immune responses are induced to regulate the host defense system. For example, Mtb infection in macrophages restricts aerobic glycolysis and IL-1β production through upregulation of miR-21. Moreover, Mtb utilizes lipid synthesis and FAO process to obtain energy and building blocks for membrane synthesis. Inhibition of FAO leads to the enhancement of mitoROS, which promote xenophagy in macrophages infected with Mtb. However, there are also controversial results that FAO is promoted by PPAR-α, which mediates anti-mycobacterial immune defense through lysosomal biogenesis and autophagy activation, *via* TFEB. The elevation of oxLDL promotes the macrophage lysosomal dysfunction, which contributes to impaired control of intracellular Mtb and host defense. Simvastatin, an oral HMG-CoA reductase inhibitor, decreases plasma cholesterol levels and exhibits host protection against Mtb through autophagy induction in monocytes.

Recent studies showed that *de novo* fatty acid synthesis (FAS) is crucial in terms of the T cell immune defense during Mtb infection, whereas FAS does not affect the innate immune responses (49). An elevated level of oxidized low-density lipoprotein (oxLDL) promotes macrophage (lysosomal) cholesterol accumulation, which leads to lysosomal dysfunction, thus impairing the control of intracellular Mtb (50). These data may explain the link between diabetes mellitus (DM) and TB through oxLDL (50). DM patients usually exhibit elevated oxLDL levels and are susceptible to TB, presumably and partly due to lysosomal dysfunction (50). In accordance with these findings, simvastatin, which reduces plasma cholesterol levels, shows protective functions against Mtb infection in several different ways (51). It inhibits intracellular Mtb growth in human peripheral blood mononuclear cells, increases the proportion of natural killer T cells, promotes production of IL-1β and IL-12p70, and activates monocyte autophagy (51). In addition, statin, the cholesterol-lowering drug, inhibits intracellular Mtb growth in human macrophages through activation of autophagy and phagosomal maturation (52). Although the precise mechanisms that induce autophagy by statins have not been fully elucidated, these findings strongly suggest that inhibitors of cholesterol synthesis and/or oxLDLs may have potential therapeutic value for TB and DM comorbidity. **Figure 1** summarizes immunometabolic regulation in macrophages during infection with Mtb, which further modulate host immunometabolism.

## AMPK-mTOR Axis Co-Regulates Autophagy and Immunometabolism

**Figure 2:**
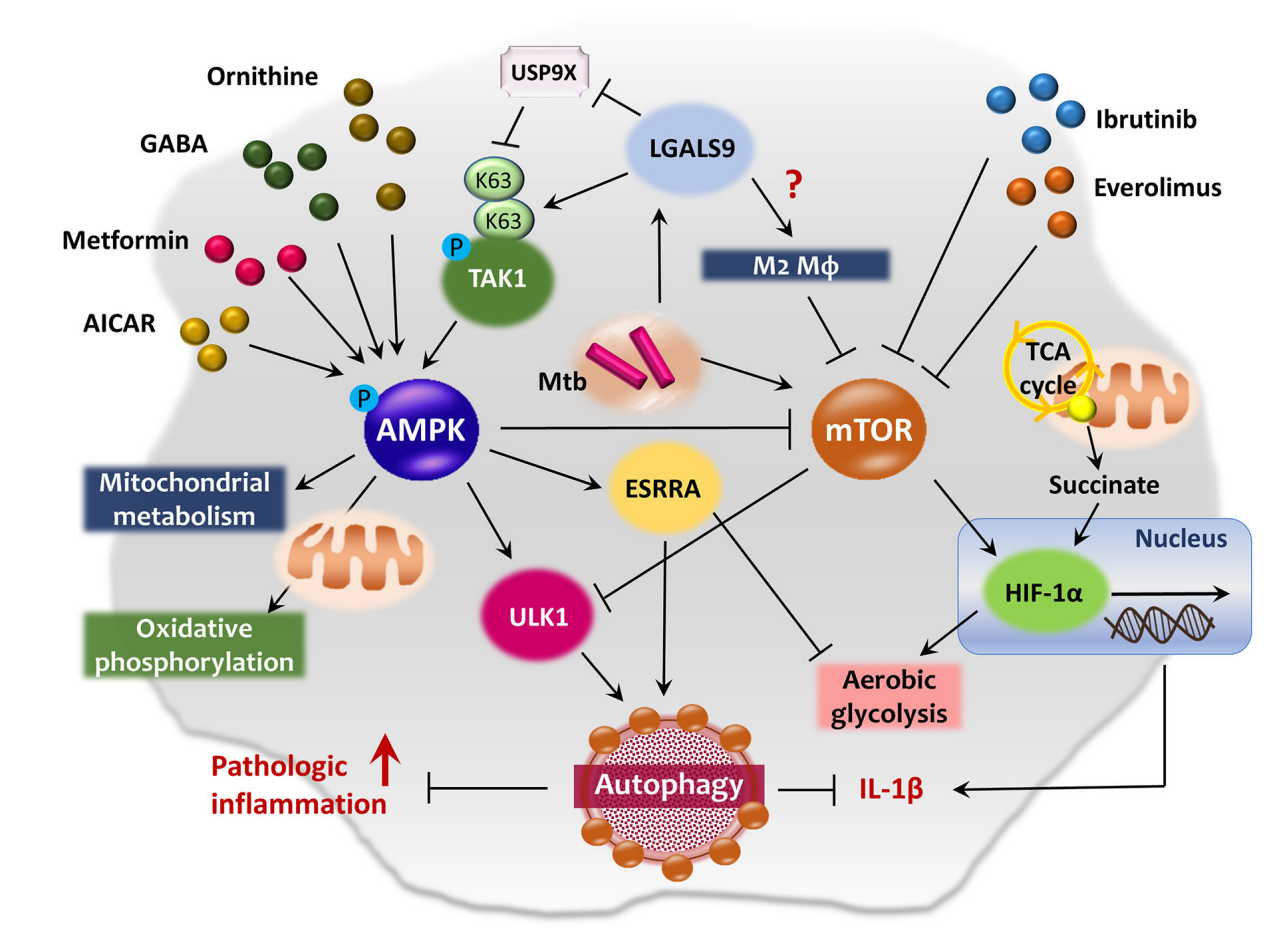
AMPK-mTOR axis in the co-regulation of autophagy and immunometabolism. AMPK pathway primarily activates mitochondrial metabolism and oxidative phosphorylation, and induces autophagy through the activation of ULK1 and inhibition of mTOR pathway. AMPK activation by AICAR, metformin, ornithine or GABA exhibits antimicrobial effects against Mtb infection in macrophages. ESRRA is one of the AMPK-downstream signaling molecule which functions as an important transcription factor of ATGs and energy metabolism. Upon lysosomal damage, cytosolic lectin LGALS9 dissociates deubiquitinase USP9X from TAK1 and promotes K63-mediated ubiquitination of TAK1, thus leading to the activation of AMPK pathway. Whereas, the mTOR pathway activation promotes aerobic glycolysis and contributes Mtb to escape from autophagic degradation in host cells by blocking ULK1 complex formation. Meanwhile, mTOR signaling is closely related to HIF-1α expression in the regulation of immunometabolism during infection and aerobic glycolysis in both normal and cancer cells. Several anticancer drugs, such as ibrutinib and everolimus, induce autophagy and repress Mtb growth *via* inhibition of mTOR pathway in macrophages. In addition, succinate, an intermediate of TCA cycle, stimulates IL-1β production *via* HIF-1α activation in LPS-exposed macrophages.

Both the AMPK and mTOR kinases are key metabolic and autophagic sensors. AMPK regulates energy metabolism and mitochondrial function (53, 54) as well as numerous biological pathways including autophagy, inflammation, and the host defense (17, 55–57). The AMPK pathway primarily activates mitochondrial metabolism, oxidative phosphorylation, and lipolysis and attenuates FAS and cholesterol biosynthesis (58, 59). AMPK enhances autophagy *via* ULK1 complex activation and mTOR complex 1 (mTORC1) inhibition (60, 61). By contrast, mTOR kinases (mTORC1 and mTORC2) suppress autophagy when energy levels are high (60). Both mTORC1 and AMPK function to integrate metabolic and autophagic signaling (60, 62), and are thus primary therapeutic targets for pulmonary TB (63, 64). The schematic overview of AMPK-mTOR axis regulating autophagy and immunometabolism is summarized in **Figure 2**.

## AMPK Regulates Autophagy and Metabolism of the Innate Host Defense System

### AMPK: A Linker of Autophagy and Immunometabolism

AMPK activation by 5-aminoimidazole-4-carboxamide ribonucleotide (AICAR) or metformin counters Mtb infection (65–67). However, our understanding of the immunometabolic regulation of AMPK-mediated autophagic activators is incomplete in the context with host defense against Mtb infection. Recent studies have shown that certain metabolites stimulate the innate host defense *via* AMPK activation. In Mtb-infected Kupffer cells, both ornithine and imidazole inhibited intracellular Mtb growth; ornithine, but not imidazole, enhanced autophagy *via* AMPK activation (68). Future studies will identify how AMPK-activating metabolites restrict Mtb growth. The metabolite and neurotransmitter gamma-aminobutyric acid (GABA) also activates AMPK, contributing to peripheral GABAergic host defenses by enhancing autophagy and phagosomal maturation during Mtb infection (69). GABA-mediated antibacterial autophagy requires the intracellular calcium influx that triggers AMPK signaling and transcriptional activation of autophagy-related genes (ATGs) including GABA type A receptor-associated protein-like 1 (GABARAPL1; an Atg8 homolog) (69).

AMPK and hypoxia-inducible factor (HIF)-1α are master regulators in the context of cancer-related aerobic glycolysis and oxidative phosphorylation. While AMPK negatively regulates both aerobic glycolysis and cellular biosynthesis, HIF-1α favors the growth advantage of cancer cells with reduced AMPK signaling (70). During Mtb infection, HIF-1α induces metabolic shift to aerobic glycolysis that amplifies macrophage activation and essentially mediates IFN-γ–dependent control of intracellular Mtb growth (71, 72). Future studies are recommended how the balance of AMPK signaling and HIF-1α pathway interplay to regulate host defense, and coordinates immunometabolism and autophagy in the context of Mtb infection.

### Downstream Signals of the AMPK Pathway

Although the cited studies strongly suggest that AMPK pathway contributes to antimicrobial host defenses by activating autophagy, we do not yet fully understand how AMPK connects with downstream signaling molecules when co-regulating autophagy and immunometabolism. Recent studies found that estrogen-related receptor α (ESRRA) served as an AMPK-downstream signaling molecule, regulating transcriptional and post-translational modification of autophagy proteins (73). The transcription factor ESRRA affects mitochondrial biogenesis, energy metabolism (74), and immunometabolic remodeling (toward oxidative phosphorylation) during the development of innate immune responses (75). It would be interesting to explore whether ESRRA regulation of immunometabolism is linked to xenophagy during Mtb infection. Moreover, glucocorticoid signaling can also activate AMPK downstream pathways, which result in the induction of autophagy/mitophagy in skeletal muscle cells (76). Given the findings that GLP-1-directed glucocorticoid action reverses metabolic inflammation and obesity in obese mice (77), it would be interesting to investigate whether glucocorticoid signaling links host autophagy to metabolic reprogramming and how it regulates host defense against Mtb infection. Future mechanistic studies will close the gaps in our understanding of the mechanisms underlying AMPK-mediated orchestration of autophagy, immunometabolism, and host defense. These efforts will facilitate the development of novel therapeutics for TB through targeting AMPK pathway.

### Upstream Signals of the AMPK Pathway

How is AMPK activated by Mtb infection? Recent studies have provided some answers. Several stimuli (including Mtb infection) trigger lysosomal membrane breaches detected by the cytosolic lectin LGALS9/galectin-9 (78). Lysosomal damage signals transduced by LGALS9 trigger dissociation of USP9X from the TAK1 complex, thus promoting K63-mediated ubiquitination of TAK1 (78). TAK1 (an upstream kinase) activates AMPK, autophagy, and antimicrobial responses to Mtb infection (79). Thus, the galectin and ubiquitin systems co-operate to activate AMPK-induced autophagy after lysosomal damage (79, 80). The cited studies did not explore the effects of LGALS9 on immunometabolism during lysosomal damage, but recent works on tumor-associated macrophages found that LGALS9 interacts with CD206 on M2 macrophages, driving angiogenesis and the production of chemokines including monocyte chemoattractant protein (MCP)-1 (81). It will be interesting to investigate immunometabolic regulation of LGALS9-AMPK pathways in the context of Mtb infection.

## The mTOR Pathway Links Autophagy and Immunometabolism

Earlier studies found that Mtb and components thereof activate mTOR and Akt pathway signaling by host phagocytes (65, 82, 83). The Akt/mTOR pathway triggers gene expression and enzyme activity, promoting aerobic glycolysis in both normal and cancer cells (84, 85). Akt/mTOR signaling is closely linked to HIF-1α expression in the context of immunometabolic regulation during infection (86), and cancer-related aerobic glycolysis and tumor progression (87). In activated CD4+ T cells, pro-inflammatory tumor necrosis factor (TNF)-α production is mediated through glycolytic activity *via* the mTOR and HIF-1α pathways (88). In lipopolysaccharide (LPS)-exposed macrophages, the tricarboxylic acid (TCA) cycle intermediate succinate stimulates IL-1β production *via* HIF-1α activation (89). Recent studies highlight that HIF-1α is required for canonical and noncanonical autophagy to impact antifungal immunity (90, 91). However, it remains elucidated whether Akt/mTOR/HIF-1α signaling coordinates aerobic glycolysis and autophagy pathway to regulate host defense against Mtb infection.

The ability of Mtb to activate the Akt/mTOR pathway blocks ULK1 complex formation by phosphorylating it, which is one of the main components required for the autophagosome generation, allowing the bacterium to escape autophagic degradation by host cells (64). Several drugs/agents inhibit mTOR pathway activation, thereby promoting antimicrobial effects during Mtb infection. For example, the anti-chronic lymphocytic leukemia drug ibrutinib inhibited Mtb growth both *in vitro* and *in vivo*, activating autophagy *via* inhibition of the BTK/Akt/mTOR pathway (92). The effects of ibrutinib on M2 polarization and immunosuppression of nurse-like cells have been described; these cells are a subset of tumor-associated macrophages found in patients with chronic lymphocytic leukemia (93). However, it is not known whether ibrutinib-mediated autophagy activation changes the energy metabolism of host macrophages. The anticancer drug everolimus inhibits mTOR, activates autophagy, and exhibits antimicrobial effects during Mtb infection (64, 94). It is well known that everolimus shifts macrophage polarization toward the M2 phenotype and downregulates the production of pro-inflammatory cytokines, thus improving the experimental outcomes of autoimmune neuritis (95). These data strongly suggest that mTOR inhibition activates antibacterial autophagy and anti-inflammatory M2-type macrophages. Indeed, mitochondrial oxidative phosphorylation and FAO are closely related to the shift to M2-like macrophages (96, 97). Thus, the mTOR-HIF-1α-mediated interplay between autophagy and immunometabolism is highly complex and require extensive molecular dissection to delineate host defensive mechanisms. An open question is whether mTOR/HIF-1α axis is a common defensive pathway through promoting glycolysis, or plays a unique protective or detrimental function directed at distinct stages of Mtb infection.

## TFEB: A Potential Coordinator of Autophagy and Metabolism During Infection

TFEB is a member of the MiT-TFE family of basic helix-loop-helix leucine-zipper transcription factors and a key regulator of lysosome biogenesis and autophagy (21, 98). Nuclear translocation of TFEB is required for the transcriptional activation of genes encoding autophagosomes and lysosomes; such translocation is regulated by mTOR-dependent phosphorylation of TFEB on Ser(211) (99). Emerging evidence suggests that TFEB is involved in mitochondrial quality control, maintaining metabolic homeostasis and mitochondrial biogenesis (100, 101). TFEB is also required for the expression of genes of mitochondrial biogenesis, FAO, and oxidative phosphorylation (102).

Several studies have suggested that the TFEB signaling axis is a promising target for autophagy-based host-directed therapeutics against Mtb. Activation of the adopted orphan nuclear receptor subfamily 1, group D, member 1 (NR1D1) by the agonist GSK4112 enhanced the autophagosomal and antimycobacterial functions of macrophages *via* TFEB activation (103). Recent studies have shown that SIRT3 is essential for development of anti-mycobacterial responses; SIRT3 activates PPAR-α–mediated TFEB nuclear translocation (104). Indeed, TFEB transcriptional activity is directly regulated by PPAR-α, a nuclear receptor involved in the regulation of metabolism, inflammation, and host defenses (48, 105, 106). However, the cited studies did not directly examine the role of TFEB in the regulation of immunometabolism in the context of mycobacterial infection. NR1D1 is a key integrator of metabolism with the circadian clock and inhibits pro-inflammatory M1 macrophages and NLRP3 inflammasome activation (107). SIRT3 and PPAR-α play crucial roles in mitochondrial quality control, oxidative phosphorylation, and FAO in various cell types (108, 109). Thus, TFEB, and its upstream signaling molecules, may orchestrate immunometabolism, autophagy, and the inflammatory response during Mtb infection.

A recent study found that immunity-related GTPase M (IRGM) and GABARAP interacted with TFEB to affect the mTOR pathway, further activating lysosomal biogenesis (110). Thus, a complicated upstream signaling network involving ATG8 proteins, IRGM, and tripartite motif family (TRIM) may perturb mTOR signaling to enhance TFEB nuclear translocation, activate lysosomal biogenesis, and trigger autophagic maturation during Mtb infection. Indeed, AMPK-mediated, lysosomal catabolic activity is mediated by MCOLN1/mucolipin 1, the lysosomal calcium channel (111), and TFEB (18, 112). Notably, the MCOLN1-TFEB pathway is essential for the host defense mediated by the disaccharide trehalose during co-infection with TB and human immunodeficiency virus (HIV) (113). Trehalose eliminates the HIV-induced impairment of xenophagic flux by enhancing nuclear translocation and activation of TFEB and MCOLN1/mucolipin 1 (113). As trehalose-mediated TFEB activation usefully inhibits atherogenic lipid accumulation by enhancing lysosomal autophagy (114, 115), it is possible that TFEB-mediated regulation of lipid metabolism is associated with the trehalose-induced antimicrobial activities in macrophages against Mtb (either alone or during co-infection with HIV). Future studies should address the immunometabolic regulation of TFEB in terms of activation of lysosomal biogenesis during Mtb infection. The pharamacological agents that facilitate host defense against Mtb infection discussed in the paper are summarized in **Table 1**.

**Table 1:** Pharmacological agents that facilitate host defense against Mtb infection by regulating autophagy and immunometabolism.

Drugs/agents	Mechanisms	Effects	References
Simvastatin	HMG-CoA reductase inhibition	Inhibits plasma cholesterol levels and intracellular Mtb growth; Increases natural killer T cells, production of IL-1β and IL-12p70, and monocyte autophagy	(51)
AICAR	AMPK activation	Induces autophagy, phagosomal maturation, and antimicrobial responses against Mtb infection	(65)
Metformin	AMPK activation	Inhibits intracellular Mtb growth and TB immunopathology; Enhances efficacy of conventional anti-TB drugs	(66, 67)
Ornithine	AMPK activation	Inhibits intracellular Mtb growth through AMPK-mediated autophagy	(68)
GABA	AMPK activation	Enhances autophagy and phagosomal maturation during Mtb infection	(69)
Ibrutinib	BTK/Akt/mTOR pathway inhibition	Activates autophagy *via* inhibition of the BTK/Akt/mTOR pathway; Inhibits Mtb growth both *in vitro* and *in vivo*	(92)
Everolimus	mTOR inhibition	Inhibits mTOR pathway; Activates autophagy and antimicrobial effects during Mtb infection	(64, 94)
GSK4112	TFEB activation *via* NR1D1 stimulation	Enhances autophagosomal and antimycobacterial functions *via* TFEB activation	(103)
Trehalose	MCOLN1-TFEB pathway activation	Kills intracellular Mtb or NTMs by activating TFEB nuclear translocation *via* MCOLN1	(113)

AICAR 5-aminoimidazole-4-carboxamide ribonucleotide; AMPK, adenosine 5′-monophosphate-activated protein kinase; Mtb, mycobacterium tuberculosis; TB, tuberculosis; GABA, gamma-aminobutyric acid; BTK, Bruton’s tyrosine kinase; mTOR, mammalian target of rapamycin; TFEB, transcription factor EB; MCOLN1, mucolipin 1.

## Conclusion

Mtb infection triggers immunometabolic remodeling of host cells. Activation of autophagy in response to metabolic and infectious stresses further shapes immunometabolism; this determines the outcome of the host defense. We have begun to understand how autophagy and immunometabolism interact within various cell types during Mtb infection. During Mtb infection, macrophage metabolic shift to aerobic glycolysis appears to contribute antimicrobial host defense through activation of M1 macrophage-mediated inflammation. However, Mtb has evolved several strategies to evade from host glycolytic flux. Modulation of lipid metabolism may activate or inhibit host antimicrobial defense in different contexts, *via* connections with autophagy. Coordination of autophagy and immunometabolic remodeling may play important roles in terms of both the effector mechanisms in play and minimization of pathological inflammation during TB infection. However, the regulators of, and mechanisms whereby, autophagy and immunometabolism combine to mount an efficient defense during Mtb infection remain poorly known. In addition, the *in vivo* relationships between autophagy and immunometabolism are difficult to predict from *in vitro* data on individual cell types.

AMPK activation and mTOR inhibition may be of therapeutic utility against human TB. The AMPK signaling is well-known for its activity to enhance antibacterial autophagy against Mtb infection. However, it also promotes mitochondrial function and oxidative phosphorylation, but not aerobic glycolysis, and shifts macrophages toward the M2 type, potentially supporting microbial growth within host cells. In addition, mTOR-HIF-α pathway activation promotes aerobic glycolysis and inflammation, inducing granuloma formation and the host innate defense early during infection. However, uncontrolled activation of inflammation may trigger extensive immunopathology and neutrophil-mediated inflammation, negatively influencing the TB-infected host. Thus, the balanced activation of AMPK-mTOR axis may contribute to the control and/or clearance of intracellular Mtb and promotes host protective immune responses during infection.

Activation of TFEB, a key transcriptional factor of autophagy/lysosomal biogenesis, is regulated by the AMPK-mTOR axis. Future studies are warranted to elucidate whether and how TFEB-mediated lipid metabolism and autophagy activation are interconnected together in the context of host defense against Mtb infection. Although much remains to be learned about the interplay between autophagy and immunometabolism by which TFEB mediates its antimicrobial effects, its potential as a therapeutic target against TB will fuel further investigations into its coordination mechanisms. Our extensive knowledge of linking autophagy with immunometabolism that drive protective anti-TB immunity will help further development of novel host-directed therapeutics against Mtb infection.

## Author Contributions

All authors contributed to the article and approved the submitted version.

## Funding

This work was supported by the National Research Foundation of Korea (NRF), grant funded by the Korea government (MSIT) (No. 2017R1A5A2015385) and by the framework of international cooperation program managed by National Research Foundation of Korea (2015K2A2A6002008).

## Conflict of Interest

The authors declare that the research was conducted in the absence of any commercial or financial relationships that could be construed as a potential conflict of interest.
